# Supplementary material for: The Intrinsically Disordered Region in the Human STN1 OB-Fold Domain Is Important for Protecting Genome Stability
Source: Biology (Basel). 2021 Sep 28;10(10):977. doi: 10.3390/biology10100977 (PMC8533325; doi:10.3390/biology10100977)
Supplement: Supplementary file 1 [file biology-10-00977-s001.zip › biology-1373580-supplementary.pptx]

## Slide 1
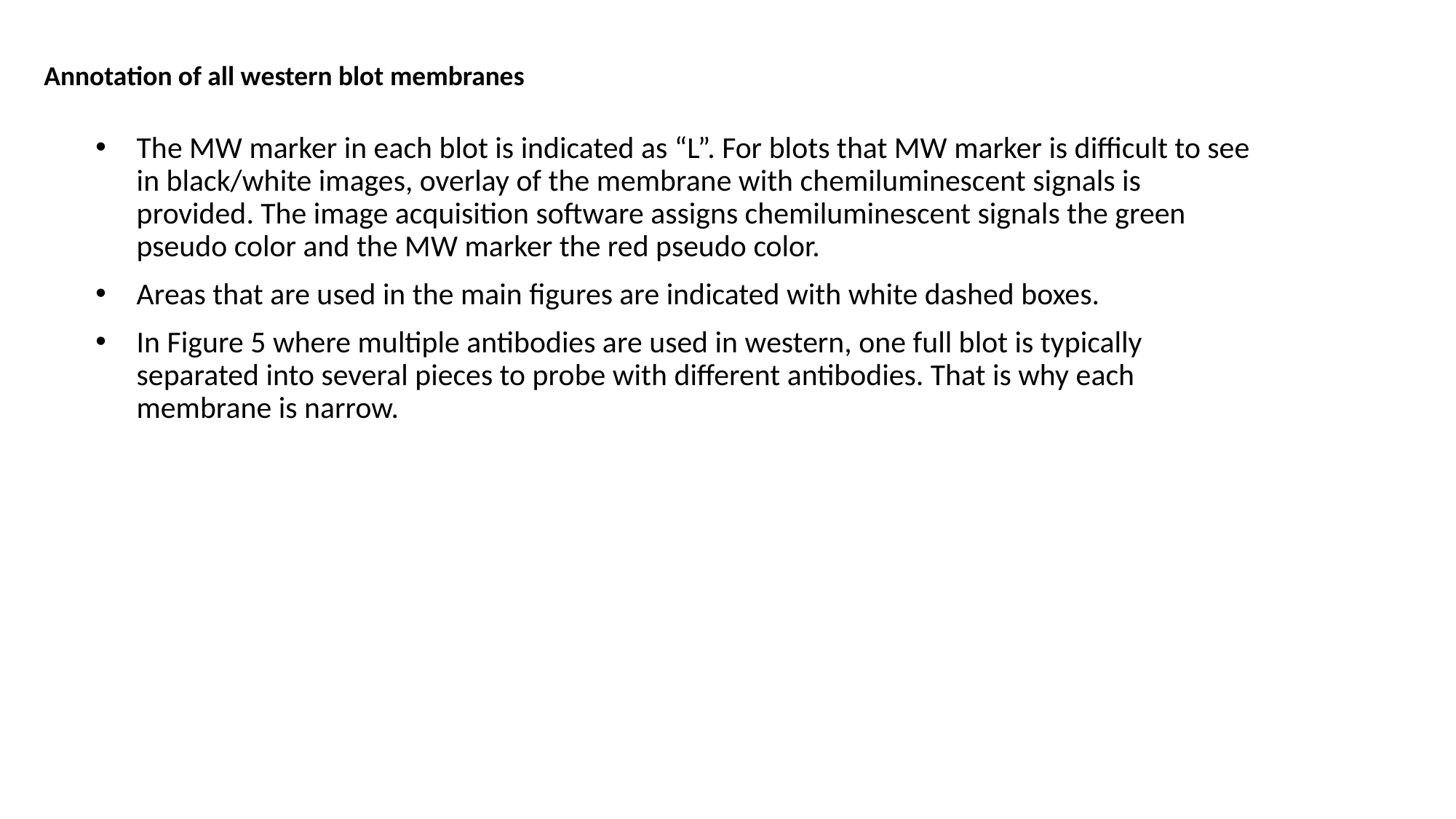

Annotation of all western blot membranes
The MW marker in each blot is indicated as “L”. For blots that MW marker is difficult to see in black/white images, overlay of the membrane with chemiluminescent signals is provided. The image acquisition software assigns chemiluminescent signals the green pseudo color and the MW marker the red pseudo color.
Areas that are used in the main figures are indicated with white dashed boxes.
In Figure 5 where multiple antibodies are used in western, one full blot is typically separated into several pieces to probe with different antibodies. That is why each membrane is narrow.

## Slide 2
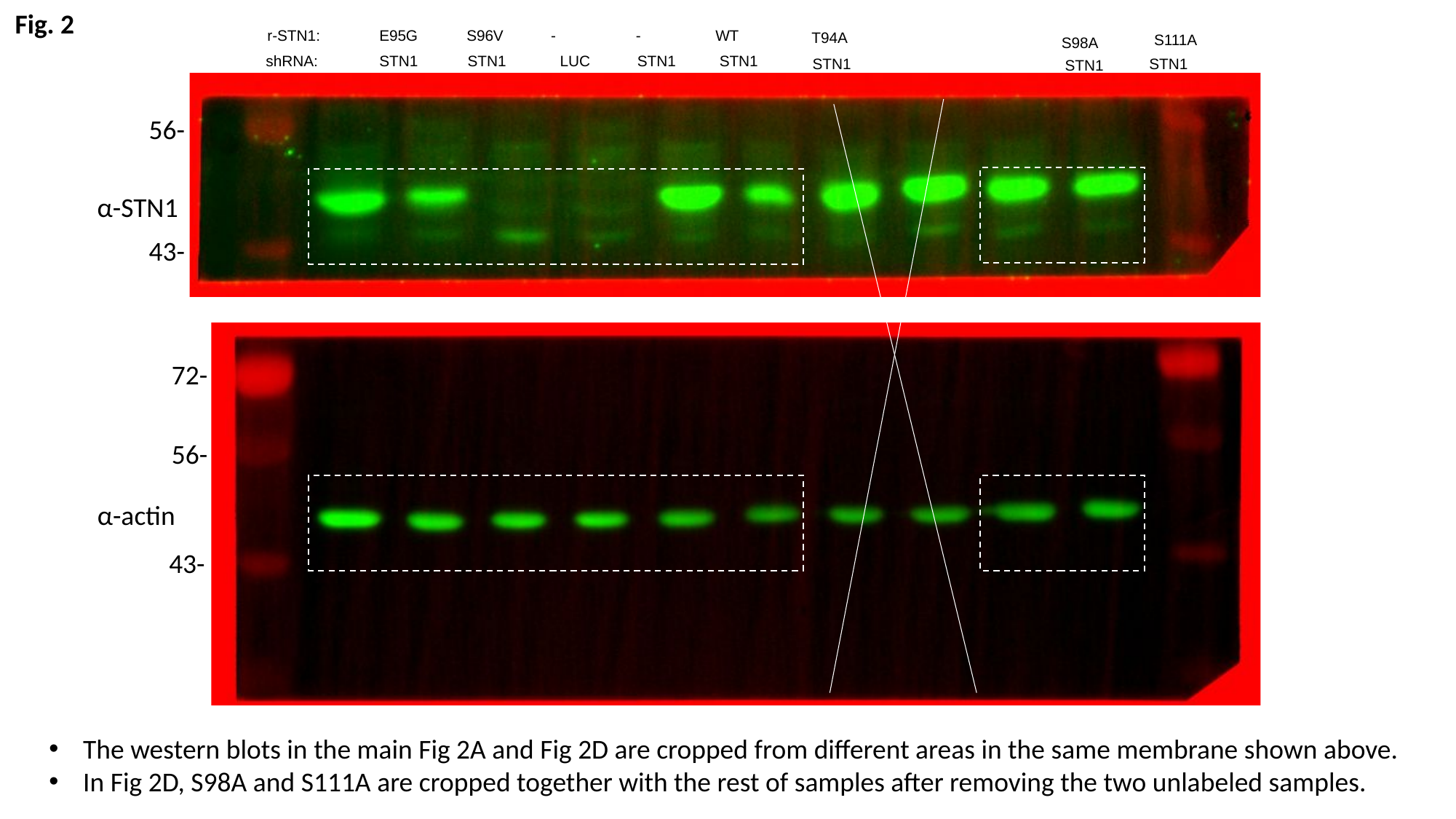

Fig. 2
r-STN1:
E95G
S96V
-
-
WT
shRNA:
STN1
STN1
LUC
STN1
STN1
T94A
S111A
S98A
STN1
STN1
STN1
56-
α-STN1
43-
72-
56-
α-actin
43-
The western blots in the main Fig 2A and Fig 2D are cropped from different areas in the same membrane shown above.
In Fig 2D, S98A and S111A are cropped together with the rest of samples after removing the two unlabeled samples.

## Slide 3
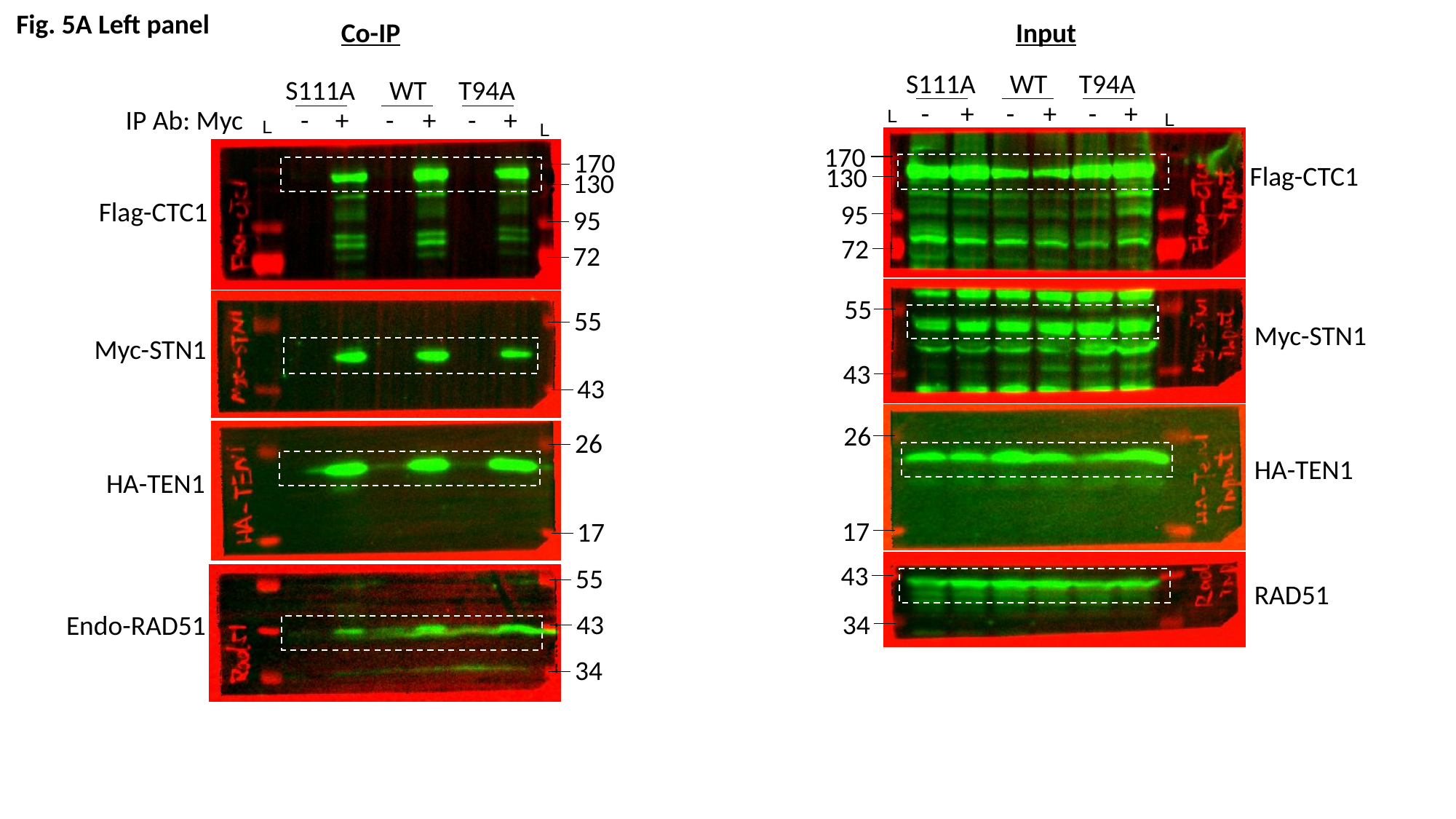

Fig. 5A Left panel
Co-IP
Input
S111A
WT
T94A
S111A
WT
T94A
-
+
-
+
-
+
IP Ab: Myc
-
+
-
+
-
+
L
L
L
L
170
170
Flag-CTC1
130
130
Flag-CTC1
95
95
72
72
55
55
Myc-STN1
Myc-STN1
43
43
26
26
HA-TEN1
HA-TEN1
17
17
43
55
RAD51
43
34
Endo-RAD51
34

## Slide 4
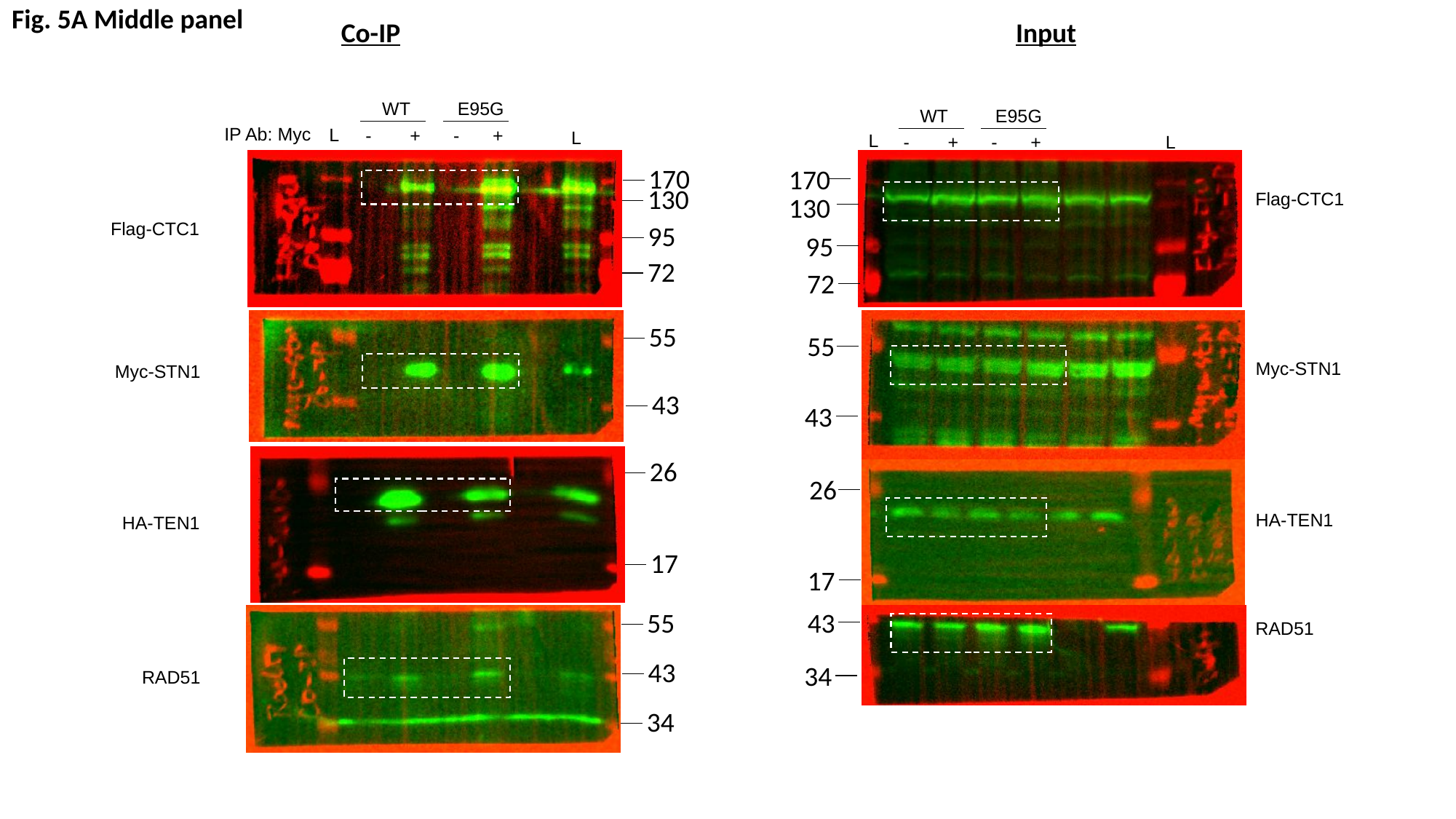

Fig. 5A Middle panel
Co-IP
Input
WT
E95G
WT
E95G
IP Ab: Myc
L
-
+
-
+
L
L
-
+
-
+
L
170
170
130
Flag-CTC1
130
Flag-CTC1
95
95
72
72
55
55
Myc-STN1
Myc-STN1
43
43
26
26
HA-TEN1
HA-TEN1
17
17
43
55
RAD51
43
34
RAD51
34

## Slide 5
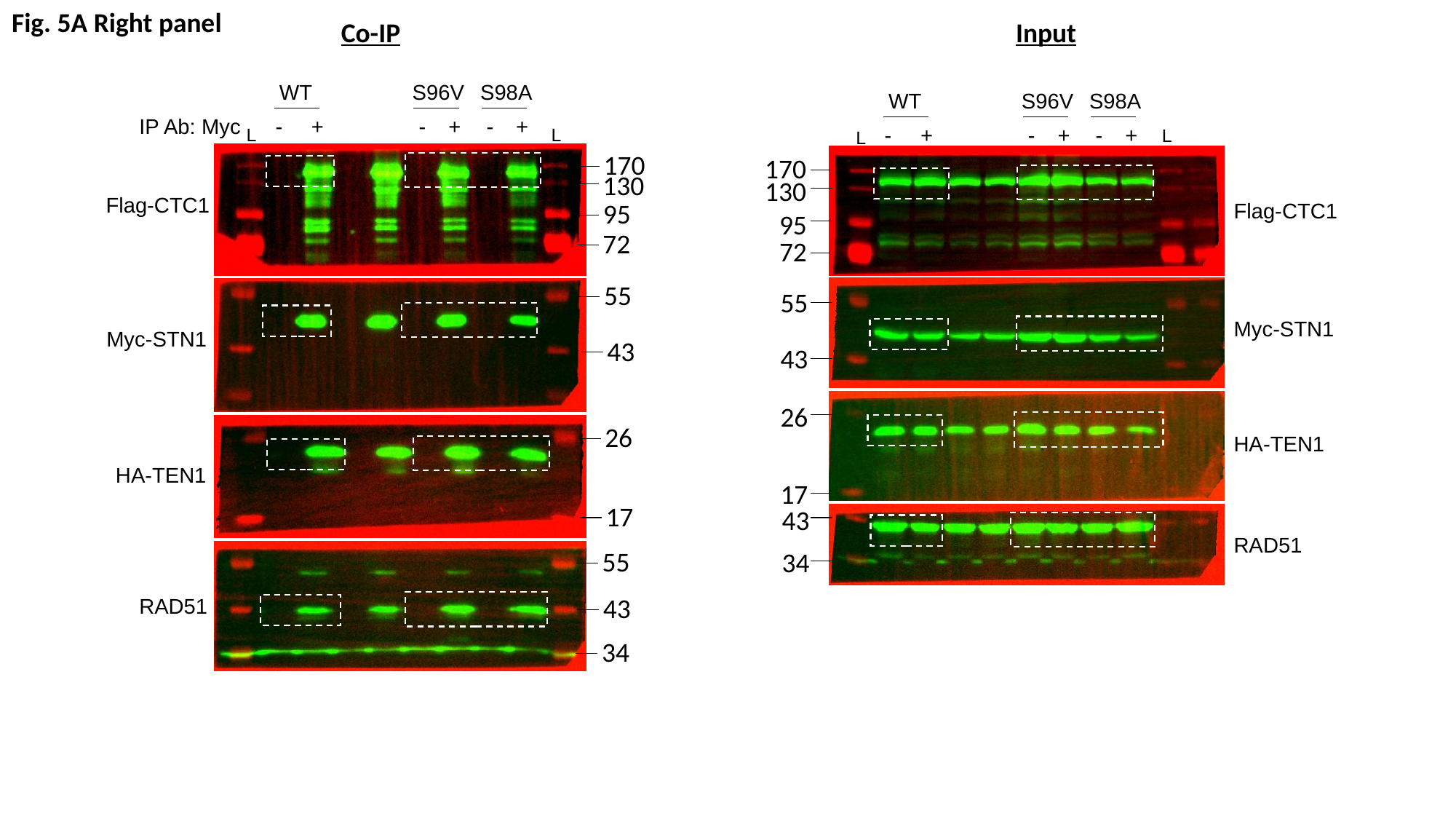

Fig. 5A Right panel
Co-IP
Input
WT
S96V
S98A
WT
S96V
S98A
IP Ab: Myc
-
+
-
+
-
+
-
+
-
+
-
+
L
L
L
L
170
170
130
130
Flag-CTC1
95
Flag-CTC1
95
72
72
55
55
Myc-STN1
Myc-STN1
43
43
26
26
HA-TEN1
HA-TEN1
17
17
43
RAD51
55
34
43
RAD51
34

## Slide 6
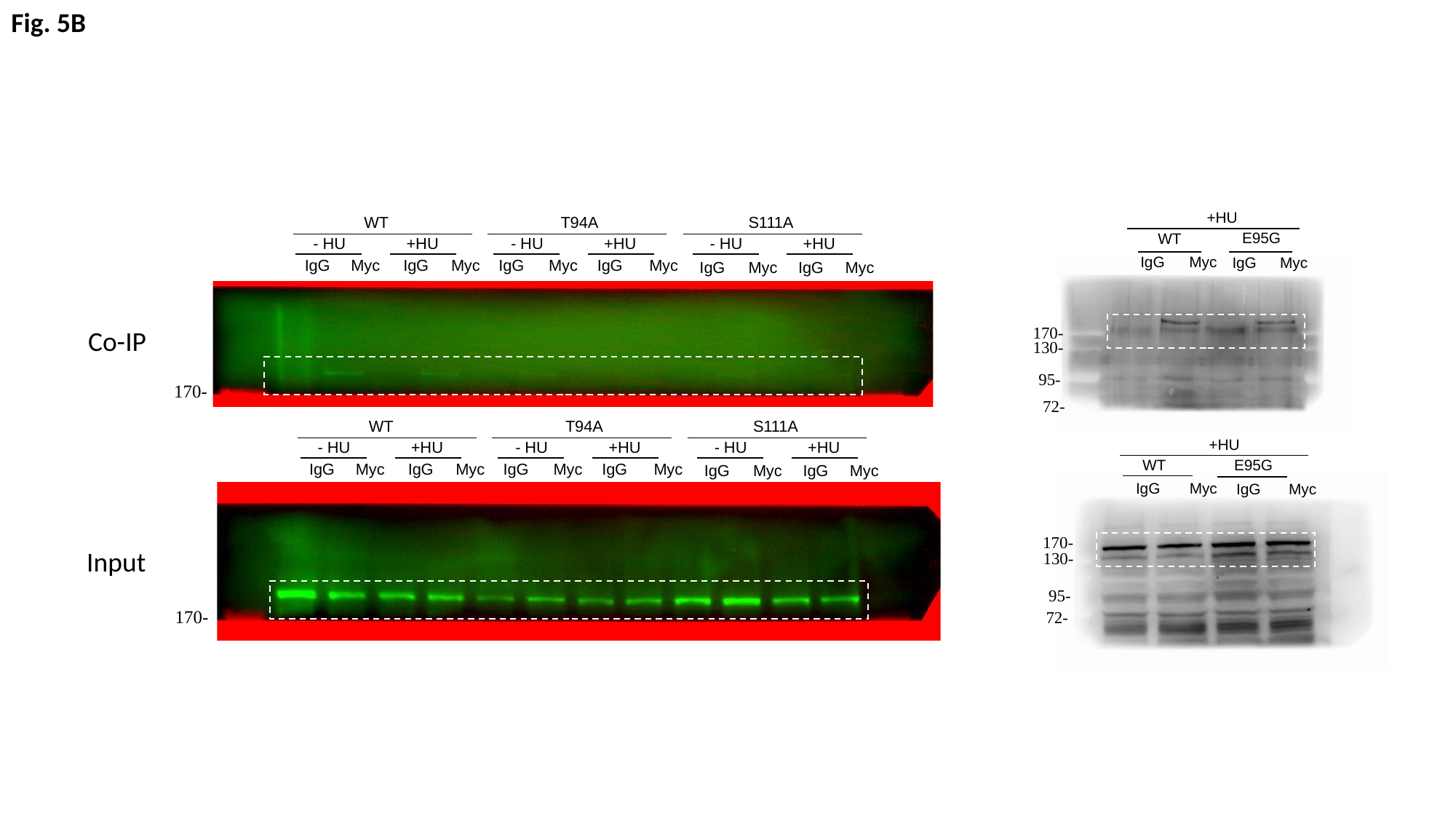

Fig. 5B
+HU
E95G
WT
IgG
Myc
IgG
Myc
WT
T94A
S111A
- HU
+HU
- HU
+HU
- HU
+HU
IgG
Myc
IgG
Myc
IgG
Myc
IgG
Myc
IgG
Myc
IgG
Myc
170-
Co-IP
130-
95-
170-
72-
WT
T94A
S111A
- HU
+HU
- HU
+HU
- HU
+HU
IgG
Myc
IgG
Myc
IgG
Myc
IgG
Myc
IgG
Myc
IgG
Myc
+HU
E95G
WT
IgG
Myc
IgG
Myc
170-
Input
130-
95-
170-
72-

## Slide 7
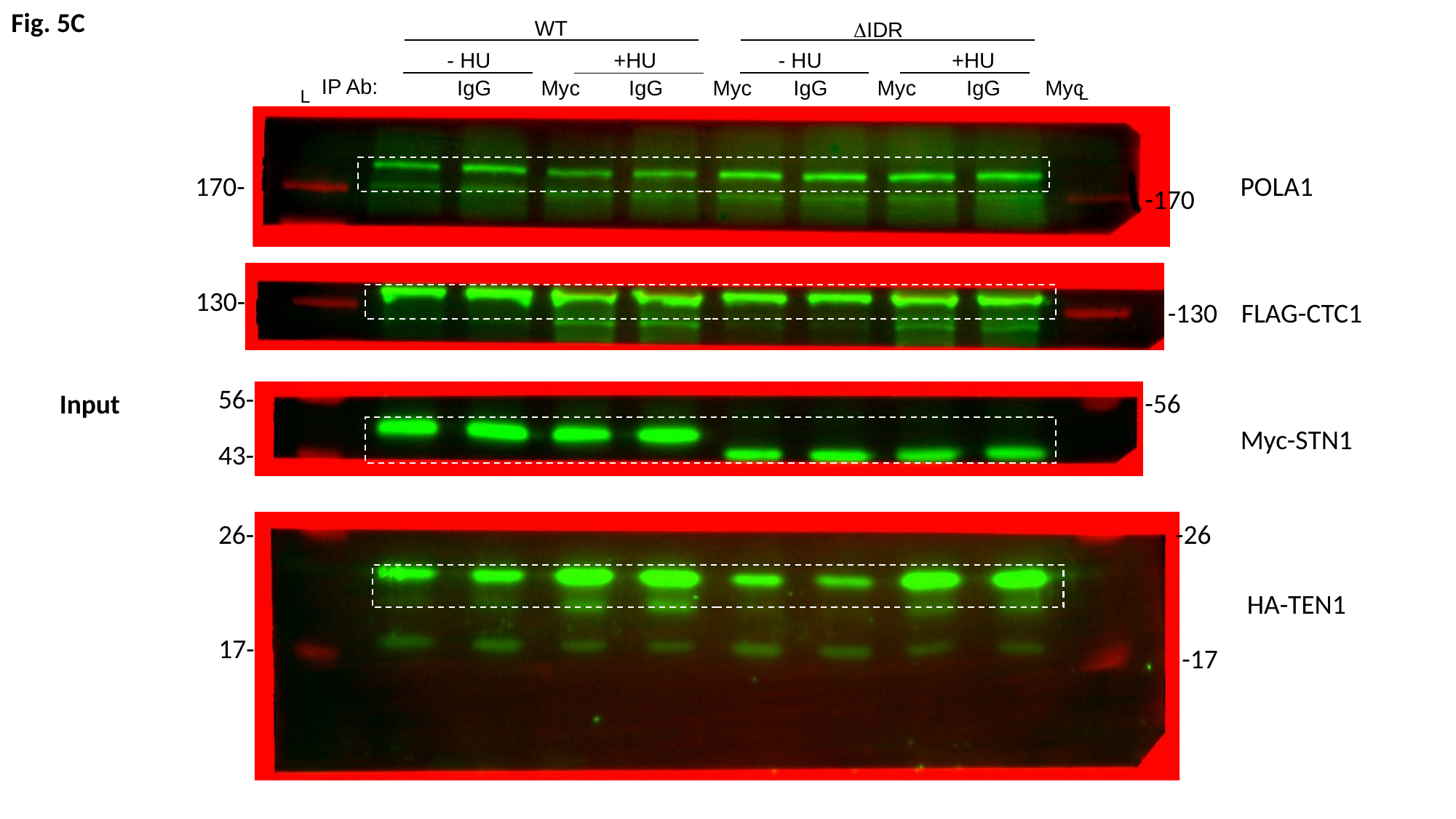

Fig. 5C
WT
DIDR
- HU
+HU
- HU
+HU
IP Ab:
IgG
Myc
IgG
Myc
IgG
Myc
IgG
Myc
L
L
170-
POLA1
-170
130-
-130
FLAG-CTC1
56-
-56
Input
Myc-STN1
43-
26-
-26
HA-TEN1
17-
-17

## Slide 8
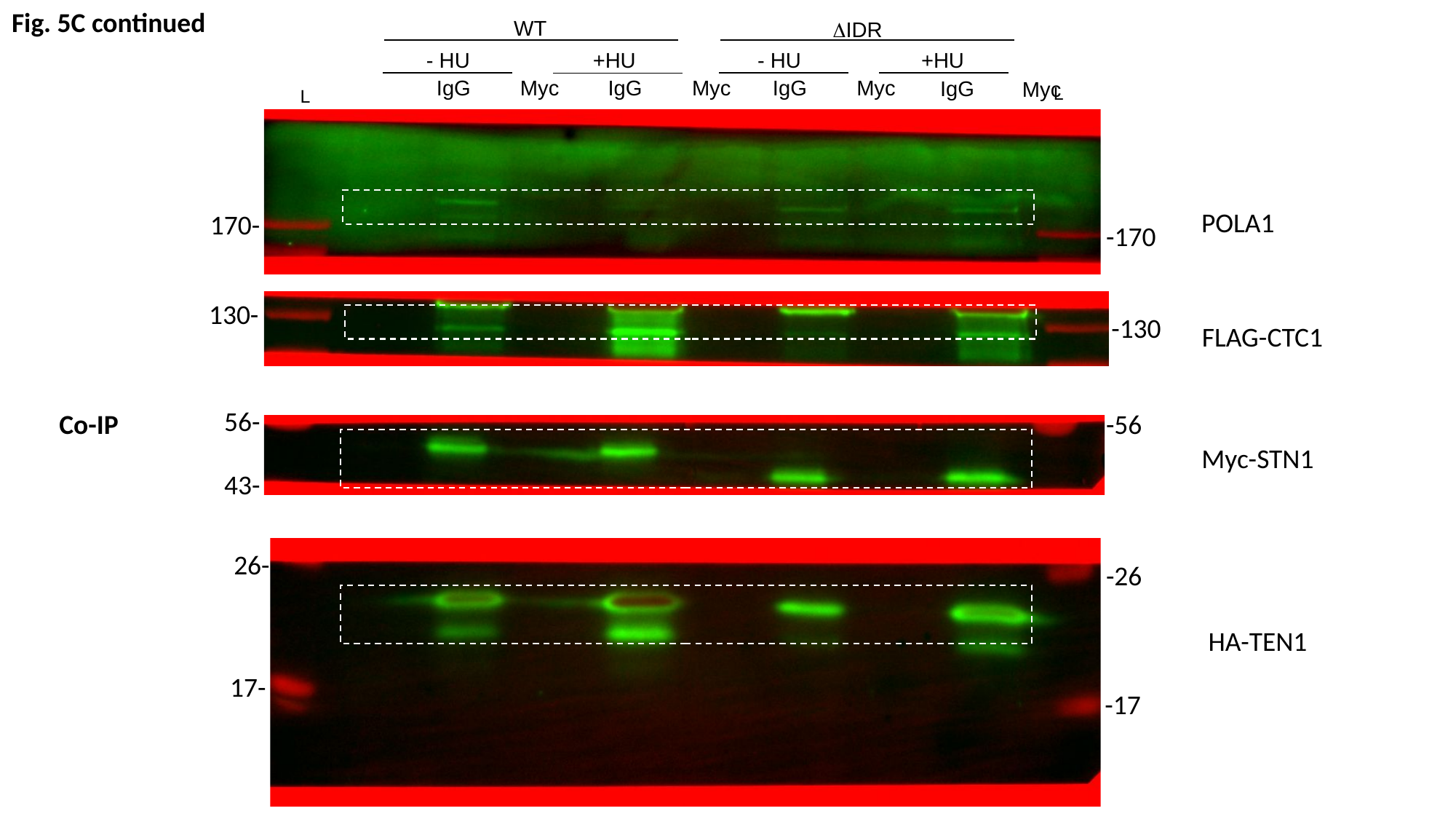

Fig. 5C continued
WT
DIDR
+HU
- HU
+HU
- HU
IgG
Myc
IgG
Myc
IgG
Myc
IgG
Myc
L
L
POLA1
170-
-170
130-
-130
FLAG-CTC1
56-
Co-IP
-56
Myc-STN1
43-
26-
-26
HA-TEN1
17-
-17
